# Lead Oxide Production in Barton Reactor—Effect of Increased Air Humidity on Lead Oxide Production Parameters

**DOI:** 10.3390/ma15144941

**Published:** 2022-07-15

**Authors:** Rafał Szela, Stanisław Małecki, Krzysztof Gargul

**Affiliations:** 1 PPUH AUTOPART Jacek Bąk Sp. z o.o., ul. Kwiatkowskiego 2a, 39-300 Mielec, Poland; rszela@autopart.pl; 2 Faculty of Non-Ferrous Metals, AGH University of Science and Technology, al. Mickiewicza 30, 30-059 Krakow, Poland; krzygar@agh.edu.pl

**Keywords:** lead-acid batteries, lead, lead oxide, Barton reactor, absorption

## Abstract

The paper presents tests of lead oxidation in a Barton reactor with a capacity of 1200 kg PbO/h, divided into two series. The first series was carried out in conditions of high humidity of the air supplied to the reactor (59–61%), and the second series in conditions of low humidity (19–21%). The study used lead of various purity levels, the main impurities of which were bismuth and silver. The obtained results show that the use of air with a humidity of about 60% in the process allows us to obtain high-quality PbO and has a positive effect on processing parameters such as the amount of lead processed and the efficiency of the process. The mentioned processing parameters significantly influence the production cost of lead oxide. The effect of lead impurities on the process of lead oxidation and the quality of the obtained product was noticed. This dependence is especially visible in the case of the process efficiency, the amount of lead processed per time unit and the amount of formed scrap. The increase in the content of impurities adversely affects each of the parameters mentioned. Optimal parameters of lead oxide regarding the expected acid absorption at the level above 16 g H_2_SO_4_/100 g PbO and the degree of oxidation at the level of 75% were obtained for the air humidity of about 60% with the content of pollutants below 100 ppm. The paper presents data on the process parameters and the relationships between them, unpublished in the literature.

## 1. Introduction

**Figure 1:**
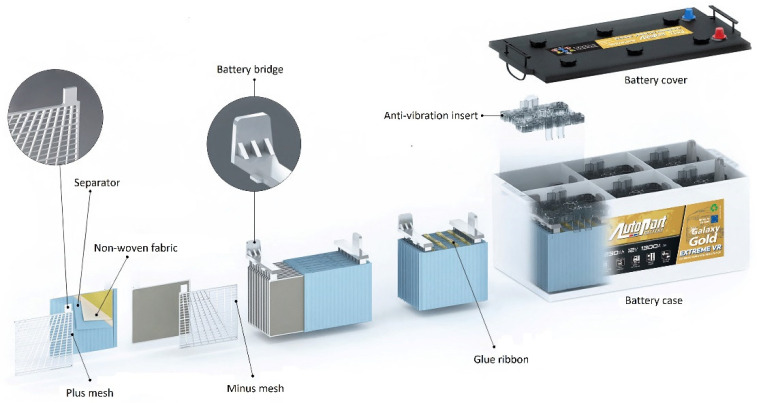
Battery construction diagram (produced by AUTOPART-Mielec, Poland).

Lead-acid batteries are the main and most common source of electricity in the automotive industry. They are also used in power systems for modern electric and hybrid cars and in various emergency power components for buildings, industrial plants, hospitals, telephone exchanges and field lighting systems [1,2,3,4,5,6]. The main factor contributing to this high prevalence worldwide is the fact that lead acid batteries are almost 100% recyclable. After the recycling process, lead can be reused to produce lead oxide and battery grids [7,8,9,10,11,12,13]. A battery is made of several interconnected cells placed in a housing (Figure 1). Each cell is made up of alternating positive and negative plates. Additionally, they are separated from each other by separators permeable to electrolyte. The vertical walls divide the battery into chambers where the battery plates do not touch the bottom. The closed battery plates are flooded with electrolyte and sealed. The battery casing is mainly made of specially selected plastic compounds. In addition, to allow the escape of battery gases such as oxygen and hydrogen produced during charging, the lids of battery blocks are equipped with specially designed channels to drain the gases [1,2,3]. The heart of each battery is its positive and negative battery plates. They consist of a battery grid and a paste placed on it. Depending on the technology of the grid production and the purpose of the battery, the produced lead grid contains various alloying components such as calcium, aluminum, tin, and antimony [14,15].

The paste applied on the grid consists mainly of lead oxide, sulfuric acid and suitably selected additives depending on the type of plate (plus/minus) and also on the type of battery (semi-traction, start stop, gel, AGM) [16].

**Figure 2:**
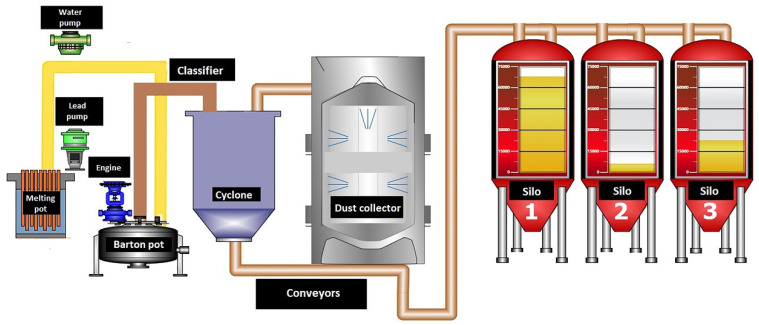
Schematic of the Barton reactor (screen from the control program in AUTOPART—Mielec, Poland).

Lead oxide used in the production of battery paste is currently produced by two methods: in a Barton reactor and in a ball mill. The Barton reactor (Figure 2) consists of a lead melting furnace and a reaction vessel equipped with a rapidly rotating stirrer. Additionally the vessel is located in a heated chamber. Lead is fed into the reactor from the furnace via a heated through. The oxidized lead oxide particles are sucked into the classifier, where the oxide is separated into grain sizes. Grains of the appropriate size are transported by conveyors to the storage silos, while the remaining grains are returned to the reactor. In order to be used for the production of battery paste, lead oxide must meet a number of technological requirements [1,2,3,5,17,18,19,20,21,22,23,24,25,26].

The physical, chemical properties and reactivity of lead oxide will vary depending on the production method, the conditions used during production and the purity of the raw materials. The thermal methods of producing lead oxide are divided into three categories depending on the temperature range. The Barton process belongs to the medium-temperature method, in which the processes range from the melting point of lead (327.5 °C) to 886 °C. The reaction occurring in the Barton reactor is exothermic and is controlled by the amount of water supplied, the amount of air supplied and the amount of lead supplied. Lead oxide production in the ball mill is carried out at temperatures below the melting point of lead. High-temperature processes are carried out above 886 °C.

The rhombic variety (β-PbO) is formed at temperatures above 510 °C, while the tetragonal variety (α-PbO) is formed during oxidation at temperatures below the melting point of lead. The lead oxide formed in the ball mill consists of 100% α-PbO, whereas the product of the Barton reactor is a mixture of both varieties (α-PbO and β-PbO). The ratio of tetragonal and rhombic varieties depends on the selected process parameters. If oxidation is carried out at temperatures of 460–470 °C then 60–70% tetragonal lead oxide (α-PbO) and 15–20% rhombic (β-PbO) is obtained; the rest is amorphous lead oxide. This ratio of lead oxide varieties is most often obtained in the production of lead oxide by the Barton method. In addition, the degree of oxidation of lead is 70–80%. The crystalline variety of lead oxide is of great importance during the formation of the battery plate and in the subsequent seasoning of the plate. The β-PbO variety favors the formation of tetrabasic lead sulfate (4BS), while the α-PbO variety produces tribasic lead sulfate (3BS). Tri-base lead sulfate leads to the formation of β-PbO_2_, which is a fine material providing a higher initial battery capacity but a lower service life. Quad-base lead sulfate leads to the formation of more α-PbO_2_. These particles improve the mechanical integrity of the positive plate and provide longer battery life.

A very important performance parameter for lead oxide is the determination of the acid absorption on its surface. This parameter provides information on the reactivity of lead oxide and the development of the surface area. The specific surface of the oxide is one of the main factors influencing the amount of absorption. The smaller and more expanded the particles are, the greater the absorption of acid. In the literature, there is only information that the absorption of sulfuric acid should be above 13.5 g H_2_SO_4_/100 g of lead oxide. The powder produced in the Barton reactor consists of spherical particles. Lead oxide, which has a lamellar structure, is produced in a ball mill and is usually preferable due to its larger specific surface area compared to spherical particles. Therefore, the oxide from the Barton reactor is subjected to a hammer mill grinding process in order to increase the specific surface area. In order to maintain the quality of lead oxide at an appropriate level, it is very important to monitor and correct the process parameters in the Barton reactor. The main parameters affecting the type, quality and efficiency of the process are: molten lead temperature, reaction temperature, chamber temperature, air draft in the reactor, reactor load, amount of water supplied to the reactor, humidity of the air entering the reactor and the type of lead and the amount of impurities present in it [1,2,3,27,28,29]. Lead impurities not only adversely affect the subsequent battery parameters but also significantly affect the production efficiency, acid absorption, bulk density of lead oxide, its specific surface area and the proportion of PbO crystalline varieties formed. In addition, they have a negative influence on the amount and type of scales formed [1,2,3,30,31].

There is very little data on process parameters in the available literature. Most of the information focuses on the parameters of the lead oxide obtained and is not related to the process parameters. This work brings a variety of information presented in the form of dependencies for various parameters that have not been published so far. This is a significant extension of knowledge about the process carried out in a Barton reactor.

## 2. Research Methodology

The research was conducted in a Barton reactor with a capacity of 1200 kg PbO/h and was divided into two stages. The first was conducted under conditions of high humidity of air supplied to the reactor (59–61%), and the second was conducted under conditions of low humidity (19–21%). Lead of different purity levels was used for the study. The content of the main elements in lead was determined using a Thermo Scientific–Waldham, MA, USA ARL 3460 OES spark spectrometer. The analysis focused on elements that significantly affect the characteristics of the material obtained, influence the parameters of the process and have an impact on the amount and form of scrap produced during the lead melting process. Key process parameters were monitored during the trials. The lead temperature (480 °C), the reaction temperature (380 °C) and the chamber temperature (490 °C) were kept constant. Other parameters such as reactor load, amount of water supplied to the reactor and reactor air draft varied depending on the process.

The reactor was thoroughly cleaned before the start of a new campaign. The oxide production process begins by heating the lead in the melting furnace to 480 °C, the reactor to 385 °C and heating the lead feed trough. When the heating of the lead trough and reactor is satisfactory, the sequence of starting the reactor by switching on the exhaust fan at minimum speed begins. The next step is to start the screw conveyors. Finally, the reactor agitator is switched on. When all equipment is ready to start, the minimum reactor load (80 A) and reactor air draft force (0.80 in H_2_O = 200 Pa) are set. The lead feed to the reactor is then started and the reaction is initiated. When the reaction temperature stabilizes at 380 °C, a test is carried out to determine the degree of lead oxidation in the resulting product. Depending on the degree of oxidation obtained, the process control parameters, such as air draft and reactor load, can be gradually changed. The parameters are changed until the correct oxidation state of 75 ± 3% is reached. Further changes in the control parameters are only possible after stabilization of the process. Depending on the type of lead and moisture the process may take from 8 to 24 h to stabilize.

The reactor load was recorded during the tests. The reactor load is an electrical parameter characterizing the rotation of the stirrer in the reactor. The maximum value of the load is 120 A. Load data were collected automatically by the process control system.

Water is supplied to the reactor to stabilize the reaction temperature and to provide oxygen molecules for the lead oxidation reaction. The amount of water is regulated in such a way as to maintain a constant reaction temperature. Too much water results in a decrease in the quality of the produced lead oxide, which manifests itself in a decrease in absorption, a decrease in the specific surface area of the oxide and, in the worst case, can lead to complete stoppage of the reaction.

The amount of lead feed is one of the main factors influencing the reaction temperature. Any increase in the rate of molten lead feed leads to a corresponding increase in the reactor temperature and subsequent changes in the lead oxide characteristics. On the other hand, too low a lead flow rate will lower the temperature of the process, which may result in the lead coagulating in the reactor and terminating the reaction. The amount of lead supplied also affects the efficiency of the process. The rate at which lead is fed into the process is automatically controlled and adjusted to the current production parameters. It depends mainly on the reaction temperature.

The force of the air draft in the reactor is responsible for the removal of lead oxide particles from the reaction space. The amount of draught influences the efficiency of the process and the quality parameters of the lead oxide such as acid absorption, bulk density, specific surface area and degree of oxidation. High values increase the yield but are associated with too early removal of lead oxide from the reactor. This results in inadequate grain size and a decrease in the degree of lead oxidation. Low thrust causes a decrease in efficiency and excessive oxidation of lead which will result in large diameter grains with a small specific surface area. In this case, the degree of lead oxidation increases and absorption decreases.

The lead oxide obtained was subjected to the following tests:

- Determination of the degree of oxidation (every 2 h) according to an internal procedure;
- Determination of bulk density using an electromagnetic volumeter;
- Determination of the absorption of sulfuric acid;
- Phase analysis (XRD).

At the end of the testing of each type of lead, the yield of oxide produced was determined and the amount and type of scrap produced was determined.

## 3. Results and Discussion

Primary and secondary lead was used. Table 1 shows the chemical composition of the lead used in the tests. Trials 1, 2 and 5 were conducted using primary lead, with low impurities, in which lead purity was above 99.99%. The rest of the tests were conducted using secondary lead, whose purity was below 99.99%.

The analysis presented shows that the main contaminants of lead are bismuth and silver.

Table 2 shows the characteristics of the tests carried out with respect to the type of lead used and the humidity of the air fed into the reactor.

Table 3 presents the numerical values of the measured process parameters and Table 4 the properties of the obtained lead oxide. These values are presented in the following Figure 3, Figure 4, Figure 5, Figure 6, Figure 7, Figure 8, Figure 9, Figure 10 and Figure 11 as a function of the amount of impurities for different humidity of air fed to the reactor.

**Figure 3:**
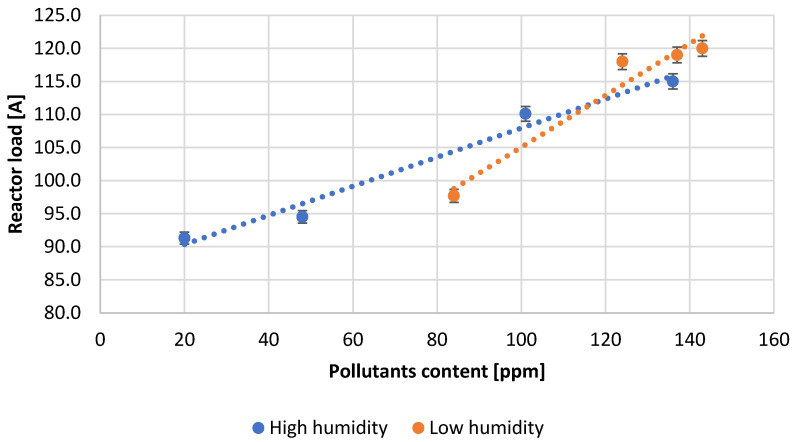
Dependence of reactor load on the pollutants content.

The dependence of the reactor load on the pollutants content allows us to notice a positive correlation for both high and low humidity of the air used (Figure 3). It should be noted that a greater influence of contaminants is observed for lower humidity.

**Figure 4:**
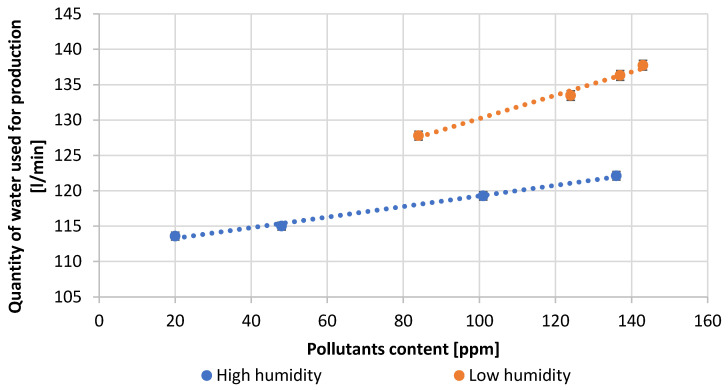
Dependence of amount of water used for production on pollutants content.

Figure 4 shows the dependence of the amount of water supplied to the reactor on the total contaminant content. The water input at high humidity conditions is much lower than at low humidity. The water molecules supplied stabilize the oxidation process and provide the oxidant needed for the reaction. As the amount of lead impurities increases, the amount of water consumed increases.

**Figure 5:**
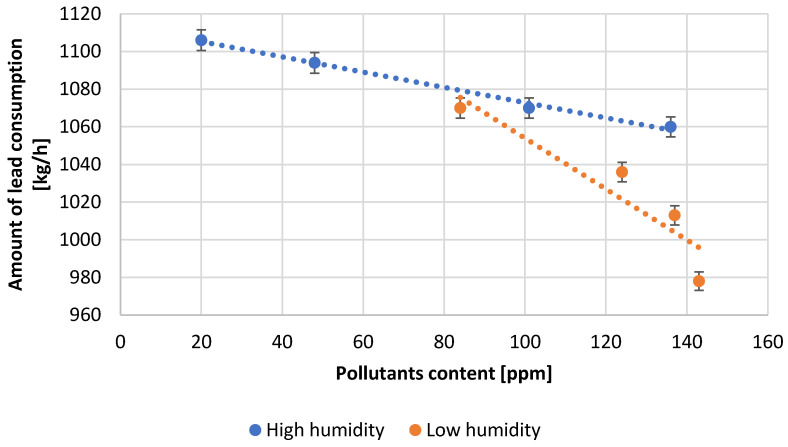
Dependence of amount of lead used on pollutants content.

Figure 5 shows the amount of lead supplied to the reactor during the production of lead oxide from lead with different levels of impurities for different humidity of the air used. Tests carried out at high air humidity allow the processing of a higher amount of lead, while the throughput decreases with increasing impurity content. This trend is also noticeable at low air humidity conditions.

**Figure 6:**
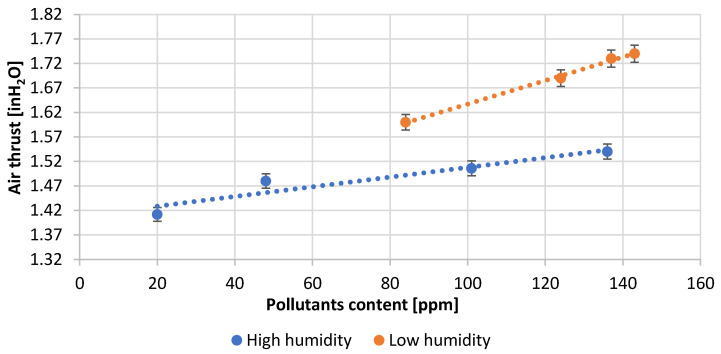
Dependence of air draft in reactor on pollutants content.

Figure 6 shows the dependence of the air draft force in the reactor on the content of impurities and on the humidity of the inlet air. An increase in the impurity content forces the use of higher air draft force for both humidities. However, higher values of air draft force are used at lower humidity. The production of lead oxide under high humidity conditions results in a more stable and efficient operation of the reactor.

**Figure 7:**
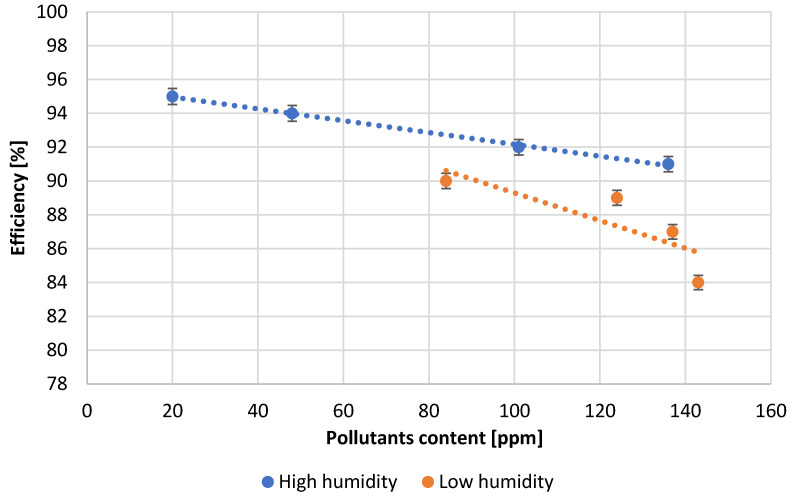
Dependence of process efficiency on pollutants content.

At the end of each measuring series, the process yield was examined. Figure 7 shows the dependence of process efficiency on the amount of impurities for samples carried out at different humidity values. The best performance is obtained for samples carried out at high humidity. A decrease in moisture content causes a decrease in process efficiency. An increase in contaminant content causes a decrease in process efficiency regardless of the humidity of the air supplied.

**Figure 8:**
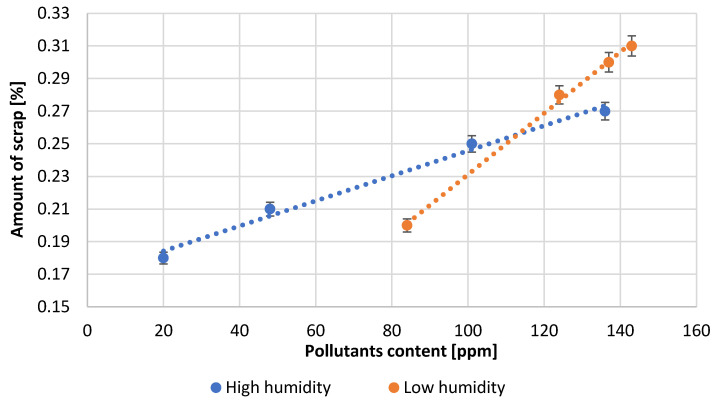
Dependence of scale formation on pollutants content.

At the end of each trial, the amount of scrap was determined as a percentage reference to the amount of lead processed. The type of scales formed was also determined (Figure 8). With an increasing amount of impurities in the lead, the scaling increases irrespective of the process conditions. At low contaminant content, dusty scrap is formed, while with increasing contaminant content, it gradually turns into doughy scrap.

**Figure 9:**
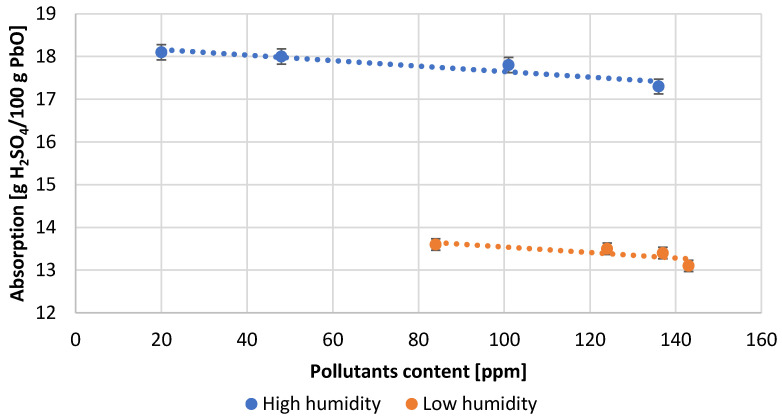
Dependence of acid absorption on pollutants content.

During each measuring cycle five samples of lead oxide were taken to determine its parameters. The obtained results were averaged. The highest values of sulfuric acid absorption were obtained under conditions of high humidity of the air used in the process (Figure 9). Under these conditions, stable operation of the reactor was observed, requiring no significant interference with other process parameters. Low air humidity results in a nearly 25% decrease in the absorption value. Under these conditions, the process was less stable and less predictable, and frequent adjustments of process parameters were needed, which had an adverse effect on the quality of the oxide obtained. It should be mentioned that irrespective of air humidity, absorption decreases slightly with increasing impurity content.

**Figure 10:**
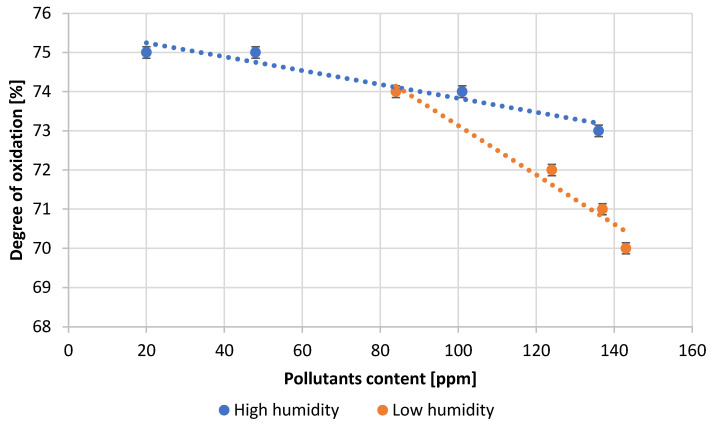
Dependence of the degree of lead oxidation on pollutants content.

The expected oxidation state values for lead oxide produced in the Barton reactor should be in the range 70–80%. The optimum value of the oxidation degree, due to the technological requirements of battery production, is 75 ± 1%. Figure 10 shows the values of the oxidation degree depending on the content of impurities for tests carried out in high and low air humidity. The results obtained for samples carried out in high humidity are within the optimum range. However, in low humidity conditions, the oxidation degree is below the expected values. Additionally, for this parameter, a decrease in its value with an increase in lead contamination is observed.

**Figure 11:**
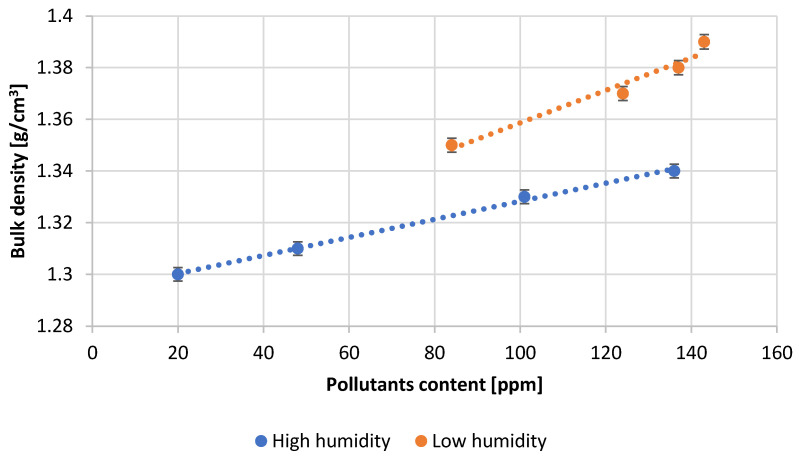
Dependence of bulk density of oxide on pollutants content.

The study (Figure 11) shows that lower bulk densities of lead oxide are obtained at high humidity conditions. Under conditions of low air humidity the bulk density of the obtained oxide increases. The density also increases with an increase in lead impurities.

The conducted research shows how important it is to choose the right parameters for lead oxide production in a Barton reactor. Changing each of them influences the quality properties of the final product. High reactor loading indicates that the lead oxidation reaction is unstable, in addition, the increase in loading may be caused by a large amount of impurities in the lead. Lead with high admixture contents can cause oxide build-up on the reactor walls. With unstable operation and large fluctuations in reaction and chamber temperature, the build-up of oxide on the walls falls off, causing a sharp increase in reactor load. The reaction is stopped and it is necessary to enter the cleaning mode of the reactor. The oxide build-up on the reactor walls is very hard and can even damage the stirrer in the reactor. Increasing the amount of lead added to the reactor increases the efficiency. Along with lead, water is added to the reactor to provide the oxidant for the reaction and to stabilize the reaction temperature. Too much lead flux can disrupt the reactor by rapidly increasing the reaction temperature. This results in a sudden increase in the degree of lead oxidation, the reaction is disrupted, the bulk density increases while the specific surface area of the oxide produced decreases. On the other hand, too much water supplied to the reactor can inhibit the lead oxidation reaction and, in the worst case, cause it to stop altogether. The increase in air draft affects the efficiency of the process and informs the size of the oxide particles formed. Too high a thrust force may remove particles from the reactor that have not reached a sufficient degree of oxidation and whose specific surface area has not been expanded to the appropriate size. Such an oxide will not meet the quality parameters in terms of absorption, degree of oxidation and bulk density. This ultimately affects battery performance parameters such as starting current and battery capacity. Low air thrust in the reactor can cause the oxidation process to be prolonged and particle size to increase, requiring increased thrust to remove such particles from the reactor. In such a case, their bulk density increases, the specific surface area decreases, and the absorption of sulfuric acid on the oxide surface decreases.

Any deviation from the optimum parameters results in a decrease in absorption. This is due to the decreasing specific surface area of the oxide produced, which is why it is so important for the process to run in a stable manner. Adjustments to the parameters must therefore be anticipated in advance in such a way as to maintain stable operation of the reactor.

**Figure 12:**
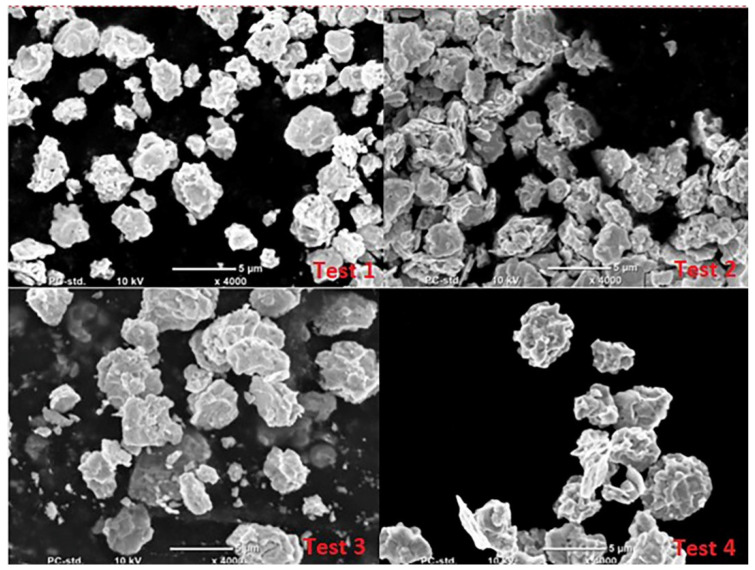
Structure of lead oxide obtained under conditions of high humidity of air fed to the reactor.

**Figure 13:**
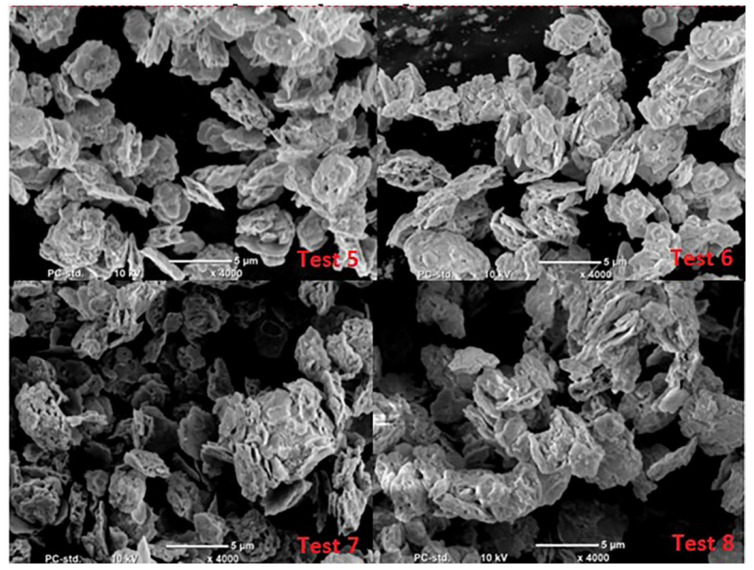
Structure of lead oxide obtained under conditions of low humidity of air fed to the reactor.

The literature analysis indicates that lead oxide grains produced in the Barton process are similar to drops or spheres, while particles produced in the process carried out in the ball mill are long, flat and similar to flakes [1,20]. Microscopic analyses of the oxides obtained in the Barton reactor indicate that they have a more developed structure, changing even at low humidity of the air fed to the reactor into a lamellar structure (Figure 12 and Figure 13). The size of oxide particles produced at high humidity is much smaller than when using low humidity air. This translates into a larger specific surface area of the oxide as indicated by the higher absorption value of sulfuric acid.

**Figure 14:**
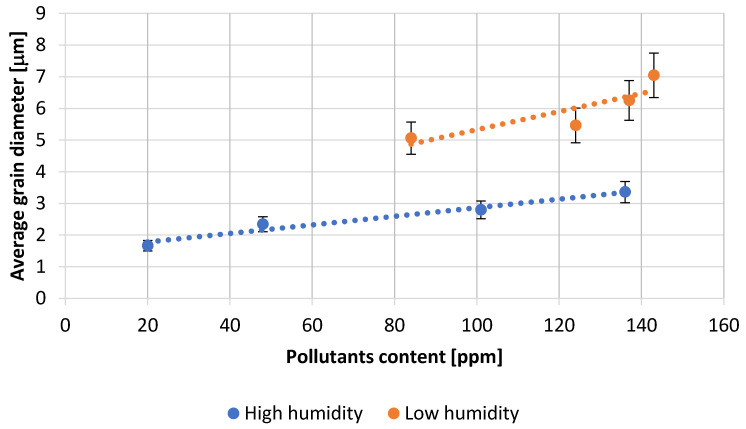
Dependence of average grain diameter of PbO on pollutants content.

The images of the grains obtained on a scanning microscope were used to determine the average grain diameter of the obtained lead oxide. The obtained dependencies are presented in Figure 14. It shows that the increase in the content of impurities influences the increase in the average size of PbO grains. Additionally, the humidity of the air fed to the reactor significantly influences the size of the grains, causing them to grow with decreasing air humidity. This is a confirmation of the dependence on the influence of the mentioned parameters on the acid absorption value. Both the grain size and absorption translate into the size of the specific surface area of the produced lead oxide.

**Figure 15:**
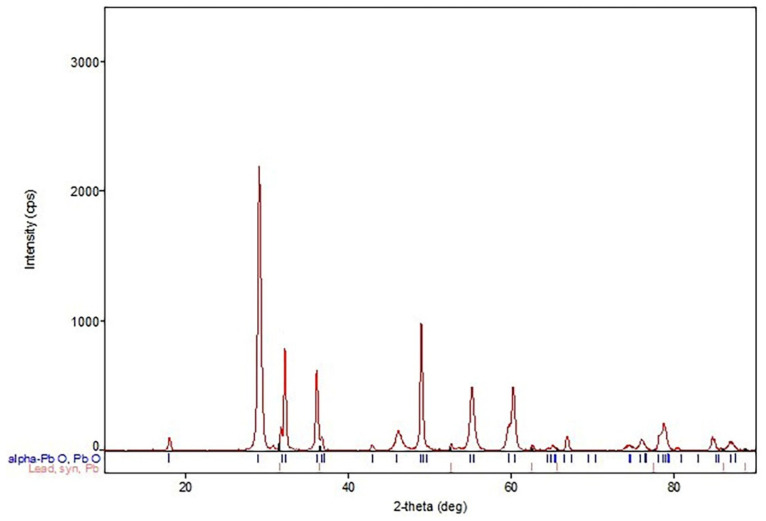
XRD analysis of lead oxide.

In addition, samples of the obtained lead oxides were subjected to X-ray phase analysis. The results of the analyses indicate an almost identical phase composition of each sample. Therefore, only one of the XRD patterns obtained is presented in Figure 15. It indicates the presence of the α-PbO phase and metallic lead. The occurrence of the β-PbO phase is difficult to state unambiguously.

## 4. Conclusions

The presented work shows the influence of many parameters on the course of lead oxidation process and properties of the obtained lead oxide. The analysis of the results of the carried out research allows us to formulate the following conclusions:

- The basic parameter deciding on the course of lead oxidation process and influencing the properties of produced lead oxide is the humidity of air supplied to the Barton reactor chamber. High humidity of approx. 60% results in high quality oxide and has a positive impact on the processing parameters such as load on the reactor, air draft, amount of processed lead and process efficiency. These processing parameters affect the cost of producing lead oxide.
- The influence of the content of lead impurities on the process of lead oxidation and the quality of the obtained product was observed. In the studied range of impurities’ content, their influence is particularly significant on the reactor load, process efficiency, the amount of lead processed per time unit and the amount of scrap produced. An increase in the content of impurities has a negative effect on each of the parameters mentioned above.
- Microscopic analysis of the lead oxide obtained confirms the conclusions formulated earlier. The process carried out under conditions of high humidity of the air fed to the reactor results in obtaining lead oxide grains of small dimensions and strongly expanded surface.
- Optimal lead oxide parameters for acid absorption expected to be above 16 g H_2_SO_4_/100 g PbO and an oxidation degree in the range of 75 ± 1% were obtained for an air humidity of about 60% at an impurity content below 100 ppm.
- Observation of the production process carried out under conditions of high humidity indicate a stable and predictable process in contrast to that carried out under low humidity.
- The results obtained in this study can be used to develop a Barton reactor control system, e.g., by means of artificial neural networks.

## Figures and Tables

**Table 1:** Content of main impurities of test lead.

Test No	Admixture Content, ppm							
	Sn	Sb	Bi	Cu	As	Ni	Ag	Other
1	0	0.2	18	0.3	0.1	0.1	1.7	0
2	0	0.2	42	0.6	0.1	0.4	2.6	2.1
3	0.2	0.4	88	0.1	0.2	0.5	10.4	1.2
4	0.1	2.9	109	1.1	0.8	0.6	20	1.5
5	0	0	75.2	0.1	0	0.3	7.7	0.5
6	1.5	1.1	115.2	2.3	0.4	0.8	2.4	0.3
7	1.1	2.4	88.9	0.9	1.4	0.8	19	22.5
8	0	4.3	110.1	1.9	0	0.4	19.6	6.7

**Table 2:** Characteristics of the tests carried out (blue color—high humidity; brown color—low humidity).

Test Number	Type of Lead	Moisture Content, [%]	Pollutants Content, [ppm]
1	Original	61	20
2	Original	59	48
3	Secondary	60	101
4	Secondary	59	136
5	Original	20	84
6	Secondary	21	124
7	Secondary	19	137
8	Secondary	19	143

**Table 3:** Process parameters for lead oxide production (blue color—high humidity; brown color—low humidity).

Test Number	Reactor Load, [A]	Quantity H_2_O, [L/min]	Amount of Pb Consumed, [kg/h]	Air Thrust, [inH_2_O]	Efficiency, [%]	Amount of Scrapes, [%]
1	91.3	113.6	1106.0	1.41	95.0	0.18
2	94.5	115.1	1094.0	1.48	94.0	0.21
3	110.1	119.3	1070.0	1.51	92.0	0.25
4	115.0	122.1	1060.0	1.54	91.0	0.27
5	97.7	127.8	1070.0	1.60	90.0	0.20
6	118.0	133.5	1036.0	1.69	89.0	0.28
7	119.0	136.3	1013.0	1.73	87.0	0.30
8	120.0	137.7	978.0	1.74	84.0	0.31

**Table 4:** Properties of the obtained product (blue color—high humidity; brown color—low humidity).

Test Number	Absorption, [g H_2_SO_4_/100 g PbO]	Degree of Oxidation, [%]	Bulk Density of PbO, [g/cm^3^]
1	18.1	75.0	1.30
2	18.0	75.0	1.31
3	17.8	74.0	1.33
4	17.3	73.0	1.34
5	13.6	74.0	1.35
6	13.5	72.0	1.37
7	13.4	71.0	1.38
8	13.1	70.0	1.39

## Data Availability

Data available on request due to restrictions eg privacy or ethical. The data presented in this study are available on request from the corresponding author.
